# Neutrophil-*Leishmania infantum* Interaction Induces Neutrophil Extracellular Traps, DAMPs, and Inflammatory Molecule Release

**DOI:** 10.1021/acsinfecdis.4c00820

**Published:** 2025-01-31

**Authors:** Paulo Ricardo Porfírio do Nascimento, Carolina Oliveira Mendes-Aguiar, Ingryd Câmara Morais, João Firmino Rodrigues Neto, Mary E. Wilson, Selma Maria Bezerra Jerônimo

**Affiliations:** † Institute of Tropical Medicine of Rio Grande do Norte, Sen. Salgado Filho Av. 3000. Lagoa Nova, 59078970 Natal, RN, Brazil; ‡ Multicamp School of Medical Sciences, Federal University of Rio Grande do Norte. Manuel Elpídio St. 610, Penedo, 59300000 Caicó, RN, Brazil; § Departments of Internal Medicine and Microbiology & Immunology, University of Iowa, 601, Hwy 6 West. 52242 Iowa City, Iowa, United States; ∥ National Institute of Science and Technology of Tropical Diseases, INCT-DT, 59078970 Natal, RN, Brazil; ⊥ Department of Biochemistry, Federal University of Rio Grande do Norte, Sen. Salgado Filho Av., 3000, Lagoa Nova, 59078970 Natal, RN, Brazil

**Keywords:** neutrophils, mitochondrial DNA, leishmaniasis

## Abstract

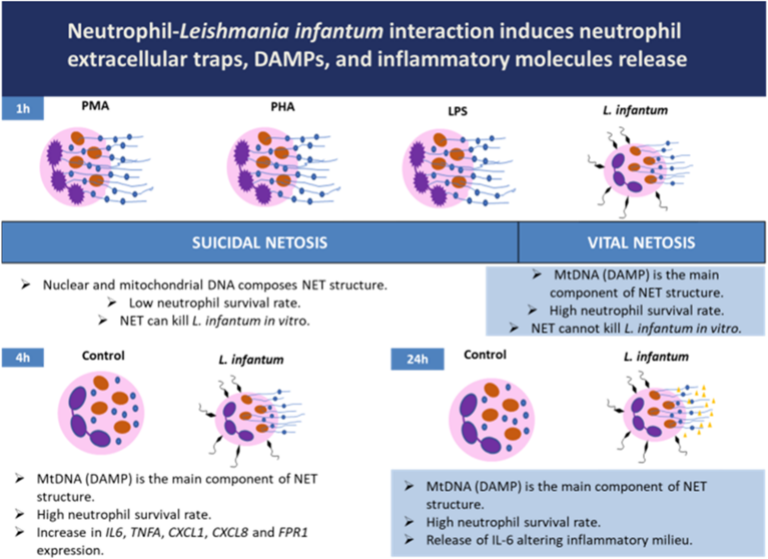

Neutrophils, the first cells to arrive at infection sites, release neutrophil extracellular traps (NETs) comprising nuclear and/or mitochondrial DNA webs decorated with proteins. Similar to other parasites, *Leishmania infantum* induces NET extrusion. However, our understanding of NET formation and neutrophil fate after NET release in a Leishmania infection context is limited. Our study aimed to determine the DNA origin of the NET scaffolds released by human neutrophils in response to chemical or *L. infantum* stimulation. Neutrophils were incubated with PMA, PHA, LPS, or *L. infantum*, followed by DNA and elastase activity quantification; additionally, we evaluated the source of DNA that composes NETs. Neutrophil viability was evaluated by annexin-V/7AAd labeling. Expression of IL6, TNFA, IL10, CXCL1, CXCL8, and FPR1 in response to the *L. infantum* interaction was assessed. Neutrophils incubated with chemicals or *L. infantum* released NETs. However, neutrophils stimulated by the chemicals showed lower viability after 1 h in comparison to neutrophils incubated with parasites. NETs from chemically stimulated neutrophils were mainly composed of nuclear DNA. Conversely, the NET induced by the parasites was of mitochondrial DNA origin and had no leishmanicidal activity. After 4 h of parasite stimulation, neutrophils peak the expression of genes such as IL6, TNFA, CXCL1, CXCL8, and FPR1. Our study demonstrates that neutrophils produce NETs after chemical or *L. infantum* exposure. Although they are not toxic to the parasite, NETs are released as danger signals. These findings support the role of neutrophils in releasing signaling molecules, which influence the inflammatory environment in which the infectious process occurs.

Neutrophils are the first immune cells to arrive at the site of infections, where they display functions that can be both antimicrobial and locally destructive, including phagocytosis of microbes, extracellular release of toxic molecules, chemokines that attract other leukocytes, and degranulation.^1^ In addition, neutrophils can respond to pathogen invasion by releasing DNA webs decorated with antimicrobial peptides including histones, cathelicidin, neutrophil elastase, cathepsin G, and myeloperoxidase. These web-like structures have been named neutrophil extracellular traps (NETs).^2^

Many studies have shown that chemical stimuli or pathogens (virus,^3^ bacteria,^4^ fungus,^5^ or protozoan^6^) can activate the release of NETs in vitro and that NETs are capable of entrapping microbes. Although apparently beneficial in the context of infectious diseases, NET release has also been implicated in pro-inflammatory disarrangements when out of strict control, leading to sterile inflammation and contributing to autoimmune disease.^6^ NET ejection can be associated with programmed cell death in a process termed NETosis, occurring with chromatin decondensation and plasma membrane rupture, leading to intracellular content extrusion.^7^ An alternative mechanism leading to NET ejection, rapid NETosis was described as a consequence of mitochondrial DNA (mtDNA) release with antimicrobial proteins attached. In the latter case, although NET markers can be detected in the extracellular environment, neutrophils continue to be viable with intact nuclear and plasma membranes.^8^

*Leishmania infantum* is the causative agent of visceral leishmaniasis (VL) in the Mediterranean Basin,^10^ Middle East, and South America.^9^ The World Health Organization estimates VL causes 100,000 cases and 10,000 human deaths per year.^10,11^ The majority of *L. infantum*-infected persons do not develop VL, and they are detected by laboratory or skin test, an evidence of controlled infection.^12^ It has become clear that neutrophils are one of the cell types affecting the interplay between innate and adaptive immune responses in leishmaniasis. Neutrophils phagocytose *L. infantum* and secrete inflammatory cytokines and chemokines, which have the capacity to activate lymphocytes and chemoattract other innate immune cells to the site of infection.^13^

Similar to other *Trypanosomatidae*,^14,15^*Leishmania spp.* have been shown to activate NET formation by human neutrophils in vitro.^16^ These webs have leishmanicidal activity, but they can be degraded by nucleotidase/nuclease enzyme secreted by the parasite, giving it some degree of resistance to NET-mediated toxicity.^17^ However, the mechanism of NET release from neutrophils in contact with *L. infantum* and the fate of the cells that extrude NETs is unknown. Furthermore, the immunological effects of infected neutrophils are not fully understood.

In this study, we aimed to evaluate the subcellular origin of DNA in the scaffold of NETs secreted by chemically or *L. infantum*-stimulated neutrophils. A secondary goal was to investigate the impact of the *Leishmania* spp. stimulus on the viability and immunomodulatory capacities of infected neutrophils. We found that nonspecific stimulation with PMA, PHA, or LPS triggered NET release, as did *L. infantum* contact. Neutrophils are short-lived cells, but *L. infantum*-stimulated neutrophils had prolonged survival to at least 24 h after infection, in contrast to chemically stimulated cells, which underwent death shortly after stimulation. The composition of NETs ejected from chemically stimulated neutrophils was primarily nuclear DNA, which exhibited leishmanicidal activity. In contrast, NETs released from *L. infantum*-stimulated neutrophils were predominantly composed of mitochondrial DNA and had no effect on leishmania viability. *L. infantum-*stimulated neutrophils also exhibited increased expression of pro-inflammatory genes *IL6*, *TNFA*, *CXCL1*, and *CXCL2*. Secreted mtDNA itself serves as a danger-associated molecular pattern (DAMP) for other cells, as stated by Itagaki and colleagues.^18^ These results highlight a potentially novel role for neutrophils during parasitic infection as a source of DAMPs, released by vital NETosis, and as a source of inflammatory and chemoattractant molecules at the site of infection.

## Results

### Chemical Stimuli or *L. infantum* Infection Activate the NET Release

**Figure 1:**
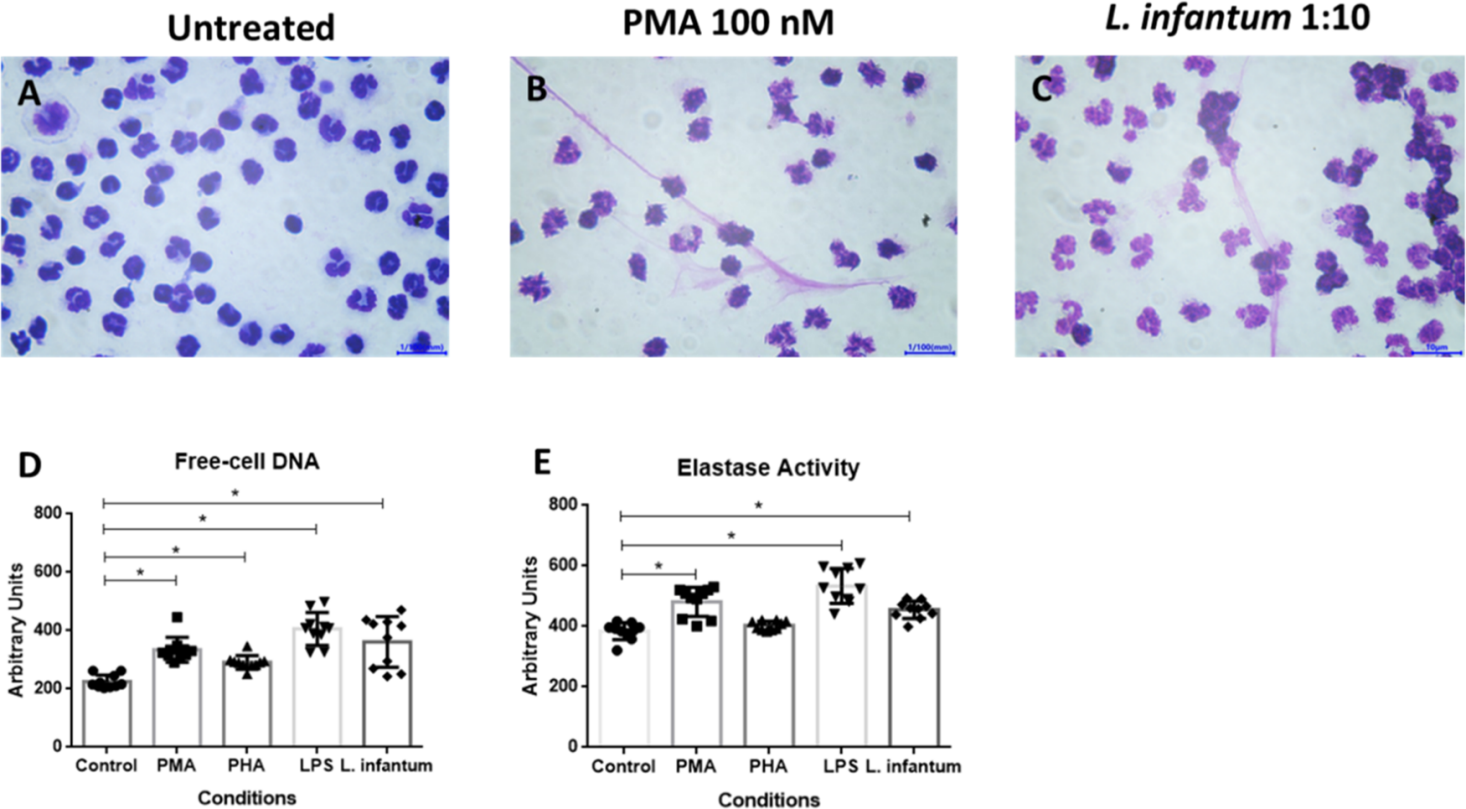
Chemical stimuli and *L. infantum* coculture can equally trigger NET release after 1 h of stimuli 100× magnification, (A–C). NET-released markers were quantified in cell culture supernatants. Cell-free DNA (D) and elastase activity (E) were estimated in cell culture supernatants as described in Experimental Section. The normality distribution of the sample values was assessed by the Kolmogorov–Smirnov test and compared means by ANOVA test. *P*-value <0.05 was considered significant*.

In response to chemical stimuli or *L. infantum* contact, neutrophils released strings of DNA-resembling webs on microscopic examinations (Figure 1A–C). To evaluate NET formation indirectly, we quantified cell-free DNA and elastase activity by fluorimetric analysis. Supernatants from untreated neutrophils had significantly lower concentrations of cell-free DNA than those stimulated with PMA, PHA, LPS, or *L. infantum* stimulation, as seen in Figure 1D. Additionally, supernatants from untreated neutrophils also exhibited significantly lower elastase activity than those stimulated with PMA, PHA, LPS, or *L. infantum* challenge, as shown in Figure 1E.

### *L. infantum* Induces NET Release without Neutrophil Killing

**Figure 2:**
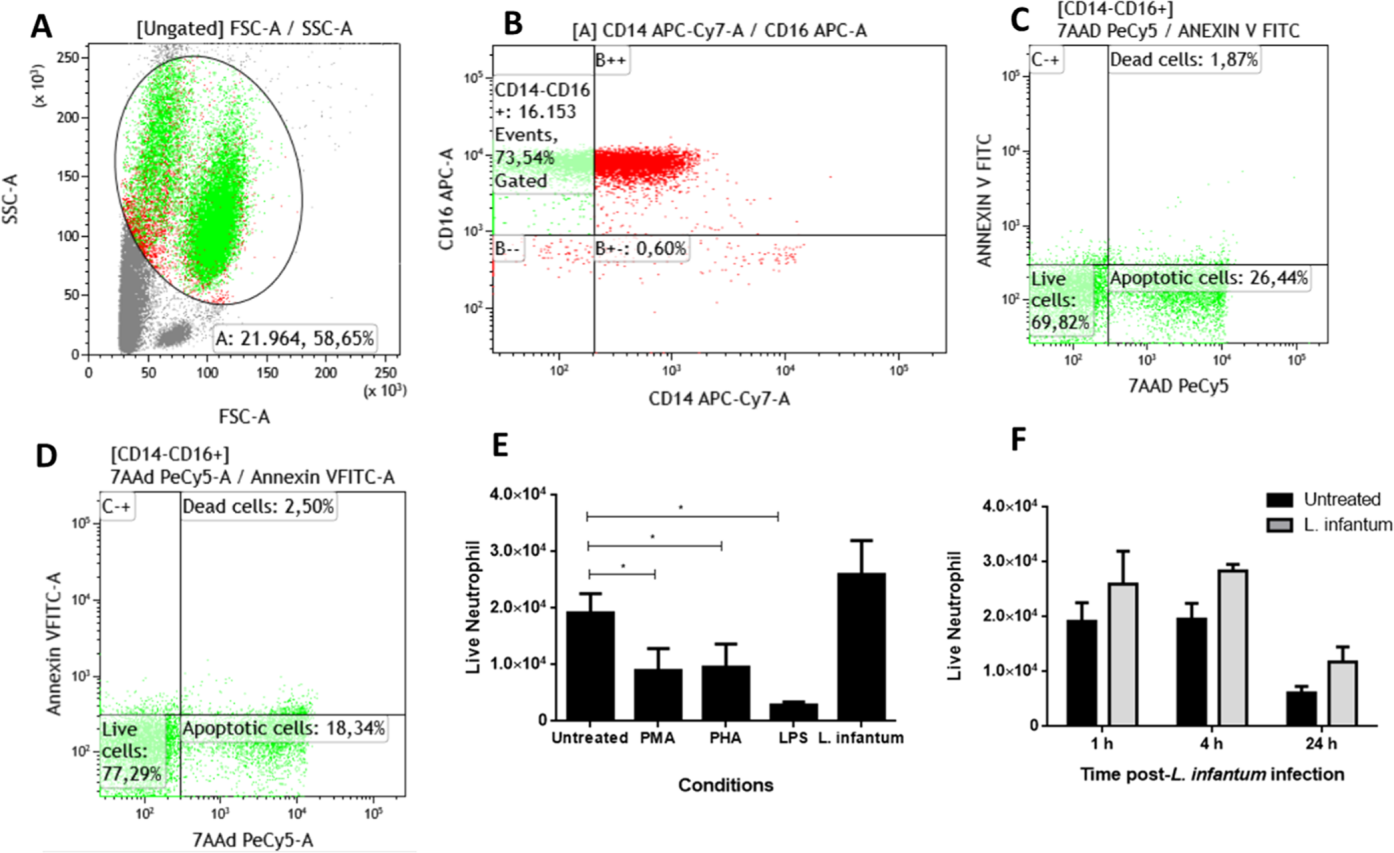
*L. infantum*-stimulated neutrophils remain viable for 1 h after parasitic challenge. Neutrophil viability was determined by flow cytometry. (A) Cell population was gathered using FSC and SSC estimated for granulocytes. (B) CD14-/CD16+ cells. (C) Neutrophils cultured with RPMI media alone (control) and *L. infantum* stimulated (D) were considered viable when 7-AAD-/annexin-V-. (E) Absolute number of recovered neutrophils (CD14-/CD16+ cells) after 1 h of experimentation. (F) Absolute number of neutrophils (7-AAD-/annexin-V-) after 1, 4, or 24 h after *L. infantum* stimulation. Figures are representative charts of 1 volunteer. We tested the normality distribution of the sample values by the Kolmogorov–Smirnov test and compared means by ANOVA test. *P*-value <0.05 was considered significant*.

In order to evaluate cell viability after the NET release, neutrophils were either chemically stimulated for 1 h or infected with *L. infantum* for 1, 4, or 24 h. Neutrophils were stained for flow cytometry analysis with anti-CD14, anti-CD16 antibodies, annexin-FITC, and 7-AAD, a double-stranded DNA-binding dye (Figure 2A–D), and the gating strategy is outlined in Figure 2. We observed that neutrophil viability was significantly reduced after 1 h of chemical stimulation with PMA, PHA, or LPS compared with untreated cells. However, *L. infantum*-stimulated neutrophils remain with high levels of viability (Figure 2E), although NET markers could be detected on cell culture supernatants.

**Figure 3:**
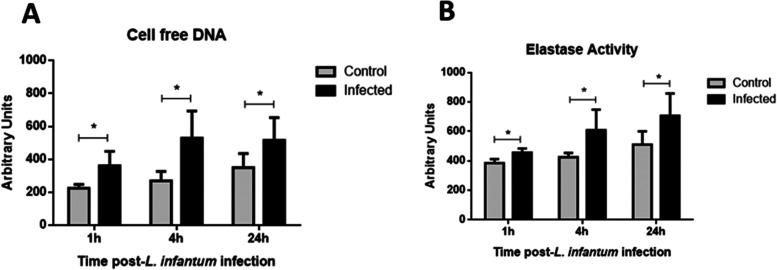
*L. infantum*-stimulated neutrophils secrete NETosis markers 1, 4, and even 24 h poststimulus. (A) Amount of cell-free DNA in cell culture supernatant after 1, 4, or 24 h poststimulation. (B) Estimation of elastase activity in cell culture supernatant after 1, 4, or 24 h poststimulus. We tested the normality distribution of the sample values by the Kolmogorov–Smirnov test and compared means by ANOVA test. *P*-value <0.05 was considered significant*.

Furthermore, *L. infantum*-stimulated neutrophils show similar survival rates of 4 and 24 h postinfection compared to untreated cells (Figure 2F). This high survival rate is accompanied by increased DNA release (Figure 3A) and elastase activity (Figure 3B). These findings instigated us to determine the source of DNA in the NETs detected.

### *L. infantum*-Stimulated Neutrophils Release Mainly Mitochondrial DNA Webs

**Figure 4:**
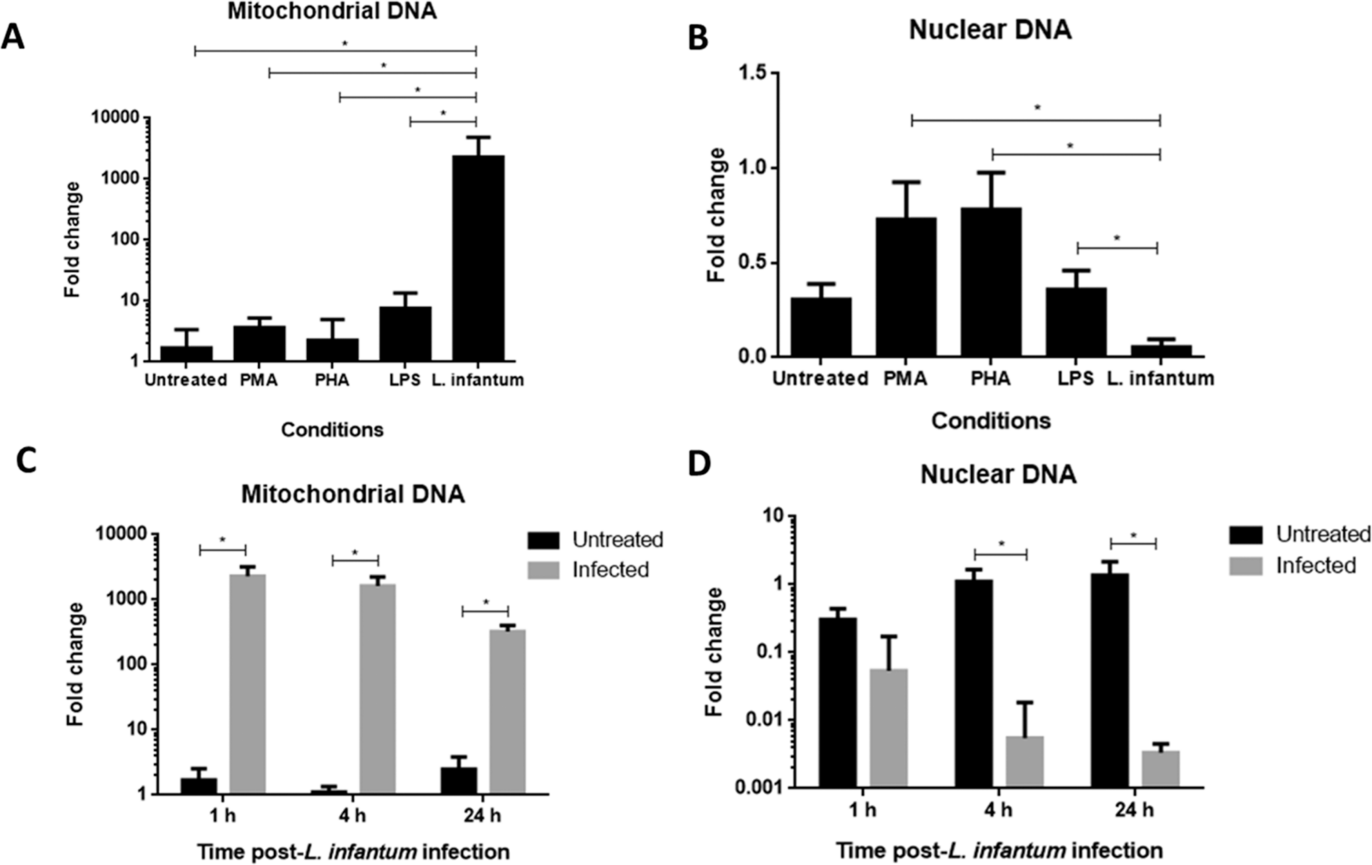
Chemically stimulated neutrophils release mitochondrial and nuclear DNA after 1 h, but *L. infantum-*cocultured neutrophils extruded majorly mtDNA. DNA obtained from cell culture supernatant was used as input for qPCR analysis using a pair of primers specific to mitochondrial or nuclear DNA. (A) Mitochondrial DNA is the main component of NETs ejected from *L. infantum*-infected neutrophils. (B) Nuclear DNA is the main component of DNA webs ejected from chemical stimulated neutrophils. (C) mtDNA was continuously released by *L. infantum*-infected neutrophils. (D) Nuclear DNA ejection was not the main source of DNA in cell culture supernatant. The normality distribution of the sample values was tested by the Kolmogorov–Smirnov test and compared means by ANOVA test. *P*-value <0.05 was considered significant.

To evaluate the source of DNA released from chemically or *L. infantum*-stimulated neutrophils, NETs obtained from cell culture supernatants were used as input for quantitative polymerase chain reaction (qPCR) analysis using a primer pair set specific for CITB, a gene present exclusively in the mitochondrial genome, or PPIA, a gene that is found only in the nuclear genome. As seen in Figure 4A, after 1 h of chemical stimulus, neutrophils released low and similar quantities of mtDNA, contrary to *L. infantum*-infected neutrophils, which extruded significantly more. We also observed that neutrophils released significantly more nuclear DNA than mitochondrial DNA after chemical stimuli, while *L. infantum*-stimulated neutrophils released negligible amounts of nuclear DNA (Figure 4B). Cells continued secreting mtDNA, significantly more than noninfected neutrophils (Figure 4C), while low levels of nuclear DNA are secreted at the same time points (Figure 4D) even at 24 h after infection.

### NETs Released by *L. infantum*-Stimulated Neutrophils Have No Antiparasite Activity

**Figure 5:**
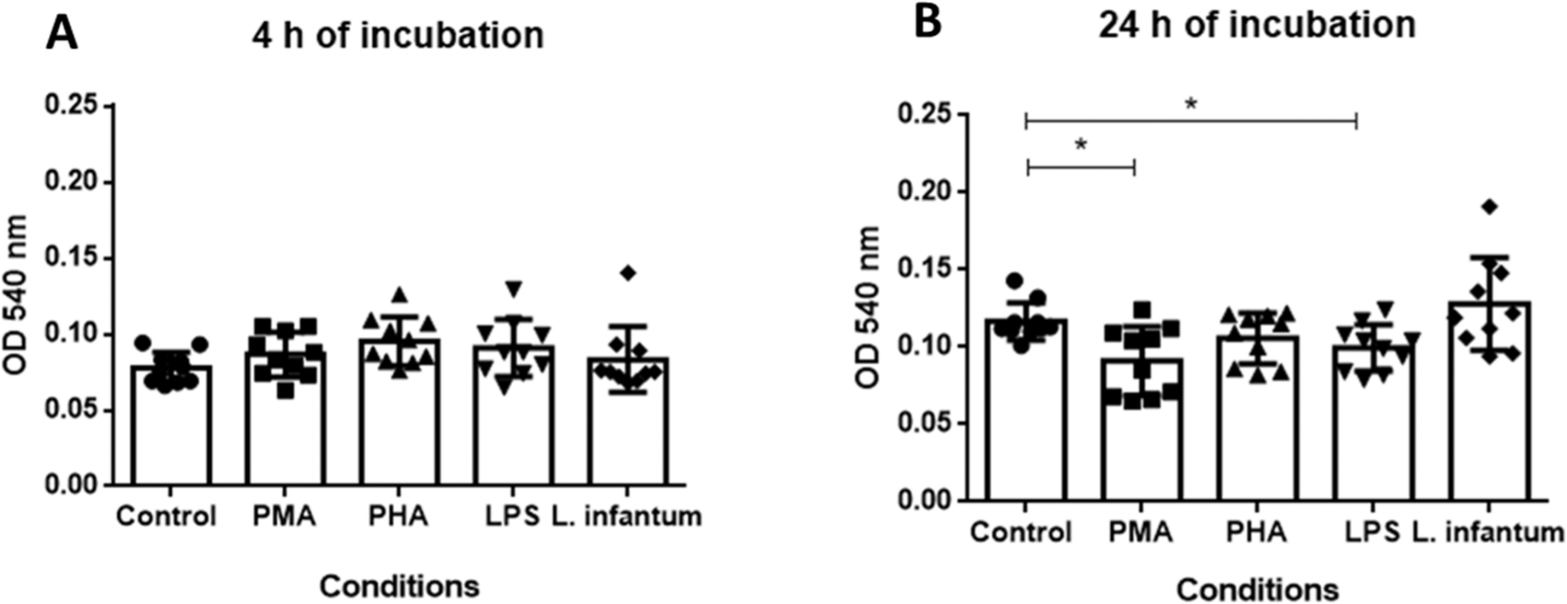
NET released from *L. infantum*-stimulated neutrophil cannot kill promastigotes in culture. (A) *L. infantum* survival rates were unaltered after 4 h of coculture with cell supernatant from neutrophils chemically stimulated or *L. infantum*-stimulated. (B) *L. infantum* survival rates were significantly decreased when cocultured with cell supernatant of neutrophils PMA and LPS stimulated compared to control. We tested the normality distribution of the sample values by Kolmogorov–Smirnov test and compared means of paired data by *t* test and multiple group analysis by ANOVA test, followed by Dunn’s post-test. *P*-value <0.05 was considered significant*.

We could not observe any effect of DNA and other compounds released by *L. infantum*-infected neutrophils on *L. infantum* survival rates after 4 h of coculture (Figure 5A). However, after 24 h of coculture with DNA and compounds released from neutrophils stimulated with PMA, the proportion of surviving *L. infantum* was reduced by 33.88%. Cell culture supernatants derived from neutrophils stimulated with PHA reduced parasite survival rates by 22.8%, while NETs derived from LPS-stimulated neutrophils reduced the *L. infantum* survival rate by 27.2%, both compared to supernatants of control. Interestingly, *L. infantum* cocultured with NETs released by stimulated neutrophils showed survival rates similar to control (Figure 5B), indicating that NETs enriched in mtDNA have no leishmanicidal activity.

### *L. infantum*-Infected Neutrophils Are a Source of Inflammatory Cytokines and Chemokines

**Figure 6:**
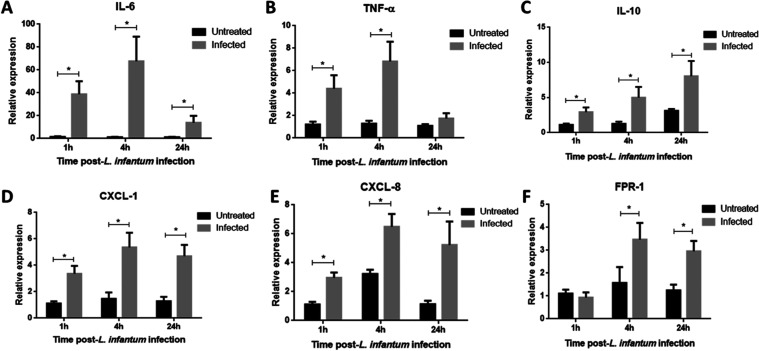
*L. infantum*-stimulated neutrophils peak gene expression of inflammatory cytokines and chemokines after 4 h of parasitic challenge. *IL6* (A) and *TNFA* (B) showed peak expression 4 h after infection compared to the uninfected control. Otherwise, *IL10* (C) showed a continuous growing pattern with a distinct transcriptional modulation from that of *IL6* and *TNFA*. Chemokine genes *CXCL1* (D) and *CXCL8* (E) also peaked their expression 4 h postinfection and remained elevated even after 24 h. (F) Expression of *FPR1*, a *N*-methylformylated peptide receptor on neutrophil surface, was significantly increased 4 h in response to *L. infantum* stimulus and remains elevated 24 h after parasitic insult. We tested the normality distribution of the sample values by the Kolmogorov–Smirnov test and compared means of paired data by *t* test. *P*-value <0.05 was considered significant*.

The expression of selected transcripts was evaluated by RT-qPCR at 3 time points after the parasite challenge (Figure 6). Transcripts were chosen because of their possible participation in the pathogenesis of VL. Expressions of all transcripts tested, including *IL6* and *TNFA* genes, were significantly more expressed in *L. infantum*-stimulated neutrophils compared to control, both showing a gene expression peak 4 h postinfection (Figure 6A,B), contrary to *IL10*, which shows a continuous growth pattern until 24 h postinfection (Figure 6C). Furthermore, *CXCL1* and *CXCL8*, genes coding for chemoattractant molecules, also show an increase in their expression pattern, peaking at 4 h postinfection (Figure 6D,E). Additionally, formyl peptide receptor-1, a cell surface receptor protein that binds actively to *N*-formylmethionine-containing peptides, is usually released by invading microorganisms or injured tissues, mainly from disrupted mitochondria. In this study, we observed a sustained increase in FPR1 expression at phils surf4 h and even 24 h post-*L. infantum* infection in neutrophils compared to control (Figure 6F).

**Figure 7:**
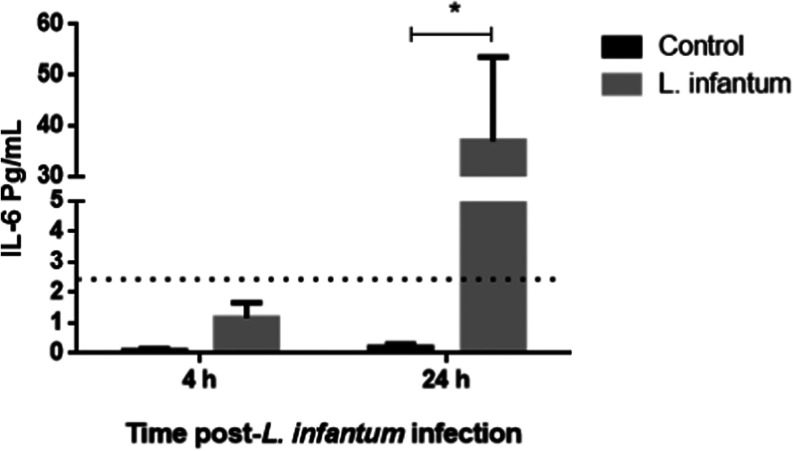
Neutrophils are the source of IL-6 after *L. infantum* infection. IL-6 concentration was determined in cell culture supernatant after 4 or 24 h postinfection. We tested the normality distribution of the sample values by the Kolmogorov–Smirnov test and compared means of paired data by *t* test. *P*-value <0.05 was considered significant*.

In order to confirm the neutrophils as a source of inflammatory cytokine during *L. infantum* infection, we estimated the concentration of IL-6 protein in cell culture supernatants of infected or uninfected neutrophils by bead flow cytometry, as IL-6 is a well-studied product of inflammatory leucocytes. We observed a significant increase in IL-6 concentration in neutrophil supernatants 24 h post-*L. infantum* stimulation (Figure 7).

## Discussion

Neutrophils are short-lived and the most abundant leukocyte present in the frontline of the immune response; they are responsible not only to phagocytose invaders but also for signaling other cells for the presence of threats by inflammatory cytokine release, chemoattractant molecules secretion, and DAMP ejection in the extracellular environment. In this study, we report that chemical stimuli such as PMA, PHA, and *Escherichia coli* LPS, as well as *L. infantum* infection, can trigger NET ejection as previously shown,^17,19−21^ but we showed, for the first time, that although infected neutrophils can release NET, they survive even 24 h after infection. Conversely, neutrophils chemically stimulated seem to activate a sort of programmed cell death, which decreases cell viability, as soon as 60 min of challenge.

Chemical compounds, such as PMA and PHA, are plant-derived organic molecules widely used as immune cell activators in experimental conditions; however, these chemicals have few or no physiological relevance, as both cannot activate any physiological process *in vivo* conditions. Otherwise, LPS isolated from *E. coli* is a molecule extensively described in the literature as an NET ejection activator, but there are divergent results. Pulze and colleagues showed detectable NET ejection with LPS 100 ng/mL in 40 min,^22^ while Remijsen and co-workers could not activate NET ejection with 10 μg/mL in 90 min after challenge,^23^ possibly due to *E. coli* strain variation and lack of manufacturer standardization procedures. In this study, this entire chemical stimulus led to the same fate for neutrophils: cell death in a short period of incubation.

Interestingly, *L. infantum*-infected neutrophils had a different fate from chemically stimulated ones. We report here that although parasitic infection can trigger NET release, it does not kill neutrophils, as we showed that 4 and 24 h after infection, the cell continues to be viable and secrete mitochondrial DNA and IL-6. Furthermore, we observed that chemically stimulated neutrophils extrude nuclear DNA in response to the insult, which may directly contribute to the low survival rates seen in these conditions. Conversely, mtDNA was the major web composition observed in NETs derived from the infected neutrophils. The chromatin structure maintenance might be partially responsible for a high survival rates observed in these cells, keeping active gene transcription, mRNA translation, and protein secretion, as well as metabolic activity, enabling the neutrophils to properly respond to infectious challenges. The case of mitochondrial DNA web release has been shown in neutrophils,^8^ eosinophils,^24^ and more recently in lymphocytes^25^ in response to chemical compound stimulation. However, in this report, we showed that the infection of neutrophils with live and viable *L. infantum* can also lead to mitochondrial DNA ejection allowing parasite multiplication, while neutrophils produce and secrete inflammatory molecules. According to Peters and colleagues, neutrophils can serve as an intermediary host cell for *L. infantum* before it is phagocyted by active macrophages, reinforcing the “Trojan horse” model of infection proposed for *Leishmania spp*.^26^

In the current study, we observed that NETs, released by neutrophils chemically stimulated by PMA and LPS, are predominantly composed of nuclear DNA and had a significant leishmanicidal effect, contrary to NETs released by *L. infantum*-infected neutrophils, which are rich in mtDNA and had no effect on parasitic survival even after 24 h of coculture. Guimarães-Costa and co-workers showed that *L. infantum* can resist the neutrophil attack by NET through the action of a 40 kDa 3′ nucleotidase/nuclease enzyme, a parasite membrane-anchored protein, which can possibly degrade DNA web scaffold and evade from killing.^17^ This finding is evidence that the NETosis mechanism triggered in response to *L. infantum* infection has immunological functions beyond parasite entrapment.

Mitochondria is an intracellular organelle with its evolutionary origin associated with a symbiotic relationship of a proteobacterium and primitive eukaryotic cell,^27^ and it plays essential roles in cell metabolism, homeostasis, and death. Possibly due to its bacterial origin, mtDNA is usually sensed as a “foreign” molecule by innate immune receptors. One of the main characteristics that points mtDNA and bacterial DNA together is the hypomethylation patterns observed in both molecules,^28^ which can readily activate pattern recognition receptors such as TLR9 and, in turn, induce an inflammatory response. It is noteworthy that *L. infantum*-infected neutrophils secrete mtDNA until 24 h after infection because this poses a new hole for these cells in the context of *Leishmania* spp. parasite infection. Neutrophils seem to act as a commander of a new avenue of cell communication, and mtDNA is the main molecular signal to advise other cells that some attack is occurring.

Beyond mitochondrial DNA, *L. infantum*-infected neutrophils presented a peak of cytokines gene expression as *IL6* and *TNFA* 4 h after infection, indicating that these molecules can have an important part in immune cell communication during an infectious process. According to Oualha and colleagues, after *L. infantum* infection, neutrophils become an important source of IL-6 and TNF-α in order to activate inflammation,^29^ as we found here. Although we could not detect significant differences in TNF-α concentration in cell culture supernatant 24 h postinfection, we observed the secretion of significant amounts of IL-6, which has a pivotal role in balancing neutrophil transit during inflammation, as it can induce the production of chemokine receptors and modulate apoptosis mechanisms.^30^ We finally observed that *L. infantum*-infected neutrophils peak the gene expression of *CXCL1* and *CXCL8* 4 h postinfection. It is well established that neutrophils can rapidly respond to chemoattractant molecules to arrive at the site of infection; however, we report here that neutrophils can act as a source of chemokine. Intriguingly, these cells can now be considered as having a paracrine action, attracting other neutrophils, as well as dendritic cells and macrophages orchestrating the first steps to the adaptive immune response activation.^31^ Lastly, we observed that neutrophils peak *FPR1* expression 4 h after infection, and this is a cell surface receptor for *N*-formylated peptides, typical of bacteria and mitochondrial products,^32^ giving strong evidence of the plasticity shown by neutrophil physiology in response to *L. infantum* infection and reinforcing the role of mitochondrial components in inflammation process.

The impact of NETosis mechanisms has been studied in the context of other parasitic infections such as Chagas’ disease, toxoplasmosis, and schistosomiasis. Souza-Rocha and colleagues showed that *Trypanosoma cruzi* and its soluble antigens can elicit NETosis in purified neutrophils in a dose-dependent way.^14^ Interestingly, a murine model of *Toxoplasma gondii* infection developed by Abdallah and co-workers pointed out that 3 different strains of the parasite can activate NETosis, and somehow, the NETs damage the parasite plasmatic membrane and reduce their capacity to infect fibroblast, so some kind of infection attenuation can be reached with this mechanism.^33^ Additionally, the contact of neutrophils with *Schistosoma japonica* eggs is sufficient to trigger NET release, and mitochondrial DNA has been detected in the extracellular milieu, mainly in the granuloma surrounding the eggs in liver tissue in mouse model experiments.^34^ This evidence indicates that NET extrusion and, particularly, mitochondrial DNA release may serve as a conserved innate immune mechanism to contain parasitic infection and signaling pathway.

In a study conducted in Ethiopia by Yizengal and colleagues, they purified neutrophils from VL patients and tested its effector mechanism activation, such as phagocytosis, degranulation, reactive oxygen species (ROS) production, and NET extrusion in response to PMA or *Leishmania donovani* infection. The authors observed that neutrophils from VL patients are not responsive to PMA or *L. donovani*, and even 30 days after treatment, the cells could not activate NETosis mechanisms.^35^ An additional study carried out in Brazil by Gardinassi and co-workers evaluated NETosis markers in the sera of VL patients and volunteers with *L. infantum* subclinical infection. This study clearly showed a molecular signature in the sera of subclinically infected volunteers composed of an increase in cell-free DNA content, MMP-9, elastase, and myeloperoxidase activity.^36^ So, the secretion of these molecules by neutrophils seems to protect the host from the development of VL. Intriguingly, this study suggests that the VL development may be directly associated with NET extrusion capacity by the neutrophils.

Taken together, our findings put neutrophils at a new level of complexity during *L. infantum* infection; it is not only the first cell line to arrive at the site of infection and phagocyte the pathogen but it also secretes NETs through a vital NETosis mechanism mainly composed of mtDNA, not necessarily in order to snare the parasite and degrade it but to signal the danger to other immune cells. Additionally, neutrophils can have a paracrine action as an important source of pro-inflammatory cytokines and chemoattractant molecules to make a possible bridge from the innate immune response based on molecular pattern recognition to adaptive immunity based on the specificity of antigen recognition by lymphocytes. The advance in the comprehension of the effector mechanisms carried out by the neutrophils in the context of *L. infantum* infection may contribute to elucidating the pathophysiology of VL and other parasitic sickness with improvement in drug development and immunobiological treatments for neglected diseases.

## Experimental Section

### Characterization of the Participants

Ten healthy adults without a history of visceral leishmaniasis were recruited, and the volunteers were 30.7 ± 5.7 years old (Table 1). Blood samples were collected and sera were checked by ELISA to exclude subclinical *L. infantum* infection.^37^ DNA were obtained from white blood cells and used as input for qPCR analysis with a set of primers specific for *L. infantum* DNA to exclude the presence of parasitic DNA circulation. Four male and 6 female blood donors were recruited. All were negative for Leishmania serology or qPCR.

**Table 1:** Characterization of Blood Donors Recruited for This Study

ID	age	gender^a^	SLA serology^b^	rK-39 serology^c^	*k*-DNA qPCR *C*_t_
1	36	M	0.221	0.235	undetectable
2	25	F	0.198	0.187	undetectable
3	30	M	0.157	0.212	undetectable
4	22	F	0.214	0.224	undetectable
5	27	F	0.193	0.241	undetectable
6	32	F	0.175	0.193	undetectable
7	28	M	0.184	0.200	undetectable
8	29	M	0.219	0.176	undetectable
9	38	F	0.155	0.195	undetectable
10	40	F	0.190	0.210	undetectable

a M: Male gender, F: Female gender.

b Cut-off value for SLA Elisa serological test was 0.275 O.D.

c Cut-off value for rK-39 Elisa serological test was 0.3 O.D.

### Neutrophil Isolation and Parasite Culture

Eight milliliters of peripheral blood were drawn from healthy volunteers in heparinized tubes. Cells were separated on a Ficoll-Hypaque density gradient (GE healthcare, Chicago, Illinois) according to Jackson et al.^38^ Neutrophils at the top of the erythrocyte pellet were collected, and erythrocytes were lysed in hypotonic buffer [(NH_4_)_2_CO_3_ 100 mM; NH_4_Cl 10 mM, 30 min, room temperature]. Neutrophils were suspended at 10^6^ cells/mL in RPMI without fetal calf serum and phenol red (Sigma-Aldrich, Darmstadt, Germany). Reference strain of *L. infantum* (Br/2000) was kindly donated by Aggeu Magalhães Institute (Fiocruz-PE) originally isolated from a VL patient in northeast Brazil and maintained in Schneider’s medium with penicillin/streptomycin 10.000 IU and 20% inactivated fetal bovine sera until experimental infection.

### Neutrophil Stimulation with Chemicals and *L. infantum* Promastigotes

Neutrophils were seeded at 10^5^ neutrophils/well in a 96-well polystyrene plate (Thermo-Fisher, Waltham, Massachusetts). Phorbol-12 myristate-13 acetate (PMA 100 nM, Sigma-Aldrich, Darmstadt, Germany), phytohemagglutinin from *Phaseolus vulgaris* (PHA 100 nM, Sigma-Aldrich, Darmstadt, Germany), lipopolysaccharide from *E. coli* (LPS 100 nM, Sigma-Aldrich, Darmstadt, Germany), or *L. infantum* (10 parasites: 1 neutrophil ratio) were added to neutrophils and were incubated with chemical stimuli for 1 h at 37 °C, 5% CO_2_. These chemical concentrations were previously determined in other studies carried out by our group. Neutrophils were cultured with *L. infantum* in early stationary growth phases for 1, 4, or 24 h.

### Neutrophil Staining and NET Marker Quantification

After incubation, neutrophils were applied to glass slides, fixed in 100% methanol, and stained with a panoptic dye to observe NET formation. Cell culture supernatants were collected and stored at −20 °C for quantification of NETs. The concentration of cell-free DNA was estimated in culture supernatant by PicoGreen reagent (485/535 nm excitation/emission; Invitrogen, Waltham, Massachusetts) and was diluted 1:300 in TE buffer and protected from light in a black opaque 96-well plate. Fluorimetric data were acquired on a Synergy LX plate reader (BioTek, Winooski, Vermont). Elastase activity was estimated using a fluorescent elastase substrate (360/455 nm excitation/emission, Sigma-Aldrich, Darmstadt, Germany), which was incubated with cell culture supernatant for 1 h, 37 °C, protected from light, and read on a Synergy LX plate reader as above.

### Flow Cytometric Assay of Neutrophil Viability

To assess the effect of chemical stimulus and *L. infantum* infection on cell viability at different time points, neutrophils were suspended in phosphate-buffered saline (PBS) containing 1:100 each of anti-CD14-APCCy7, anti-CD16-PECy7, antiannexin-FITC, and the double-stranded DNA dye 7-AAD and protected from light (antibodies and dye from BD Biosciences, Franklin Lakes, New Jersey). After 30 min at 4 °C, flow cytometry data were acquired on an FACS Canto II Flow cytometer (BD Biosciences, Franklin Lakes, New Jersey). Data were analyzed using Kaluza Analysis software v2.0 (Beckman-Coulter, Brea, California).

### MTT Assay of *L. infantum* Viability

In order to examine the effect of NETs from different sources on *L. infantum* viability, 10^5^*L. infantum* were incubated in 100 μL/well of cell culture supernatants from differentially treated neutrophils. After 4 or 24 h at 28 °C, cells were pelleted by centrifugation, and supernatants were replaced with 100 μL of MTT (1 mg/mL, Sigma-Aldrich, Darmstadt, Germany). After an additional 4 h at 28 °C, the cells were centrifuged, and dimethyl sulfoxide (DMSO) was added to solubilize formazan crystals (P. A., Sigma-Aldrich, Darmstadt, Germany). Colorimetric data were acquired on a Synergy LX plate reader at 540 nm.

### qPCR Assay of Mitochondrial and Nuclear DNA

To determine the source of DNA that composed of NETs from neutrophils under different conditions, cell culture supernatants were collected, DNA was extracted using BioPur mini spin plus, according to the manufacturer’s instructions (Pinhais, Paraná, Brazil), and 80 ng was analyzed by qPCR experiment for the mitochondrial gene Cytochrome *b* [CITB (FW-5′ GTGATTGGCTTAGTGGGCG-3′; RV-5′ AGGCGTCCTTGCCCTATTAC-3′)] and for peptidyl-prolyl-isomerase A [PPIA (FW-5′ CAAGACTGAGATGCACAAGTG-3′; RV-5′ GTGGCGGAT TTGATCATTTGG-3′)], a gene that is unique to the nuclear gene. qPCR reactions were performed using the Power up SYBR green reagent (Thermo-Fisher, Waltham, Massachusetts) according to the manufacturer’s instructions. Thermal cycling was, according to the manufacturer’s instructions, an initial denaturation step at 95 °C for 10 min, followed by 40 cycles with 15 s denaturation step and 60 s in annealing/extension at 65 °C; after the amplification step, a melting curve analysis was carried out to confirm the amplification specificity in a QuantStudio 3 qPCR system (Thermo-Fisher, Waltham, Massachusetts). (Thermo-Fisher, Waltham, Massachusetts). Comparative estimations of the amounts of mitochondrial or nuclear DNA were done using the ΔΔ*C*_t_ method as outlined in Livak et al.^39^*C*_t_ values for the nucleic gene were used as a reference for ΔΔ*C*_t_ analysis.

### Expression of Inflammatory Genes

In order to assess the effect of *L. infantum* infection on neutrophil inflammatory response, we used premade TaqMan assays for human IL6 (Hs00985639_m1), IL10 (Hs00961622_m1), TNFA (Hs00174128_m1), CXCL1 (Hs00605382_gH), CXCL8 (Hs00174103_m1), and FPR1 (Hs0423526_s1). The TaqMan assays and TaqMan Universal Master Mix were obtained from Thermo-Fisher, Waltham, Massachusetts. RNA was collected from neutrophil cultures after 1, 4, or 24 h of infection, and RNA was collected in Trizol reagent (Invitrogen), followed by the extraction of total RNA, treatment with DNase, resuspension in nuclease-free water (Invitrogen), and quantification on a NanoDrop 2000 spectrophotometer (Thermo-Fisher, Waltham, Massachusetts). cDNA was generated using a high-capacity cDNA kit (Thermo-Fisher, Waltham, Massachusetts) according to the manufacturer’s instructions. qPCR reactions in 10 μL were performed on a QuantStudio 3 Real-Time PCR System (Thermo-Fisher, Waltham, Massachusetts). Relative gene expression was analyzed by the ΔΔ*C*_t_ method^39^ using *Actin* (Hs99999901_s1) as a housekeeping gene.

### Cytokine Quantification by Cytometric Bead Array (CBA)

IL-2, IL-4, IL-6, IL-10, IL-17A, IFN-γ, and TNF-α were quantified in supernatants from neutrophils infected for 4 or 24 h with *L. infantum*. A human Th1, Th2, and Th17 cytokine bead kit from BD Biosciences (Franklin Lakes, New Jersey) was used according to the manufacturer’s instructions. Data were acquired on a FCAS Canto II Flow cytometer and analyzed using FCAP version 3 software (BD Biosciences).

### Statistical Analysis

Data were first analyzed for normality using the Kolmogorov–Smirnov test. Paired data were compared by *t* test or Mann–Whitney test. Analyses were performed with ANOVA or Kruskal–Wallis, with Dunn’s correction for multiple comparisons, using GraphPad Prism 6 software. Data from 3 independent experiments were analyzed collectively. A *p*-value of <0.05 was considered statistically significant.

### Ethical Considerations

The human study protocol was reviewed and approved by the Universidade Federal do Rio Grande do Norte Ethics Research Committee (CAAE14070213.3.0000.5537). Procedures followed the ethical principles of the Declaration of Helsinki. Consenting volunteer blood donors provided signed informed consent prior to the blood draw.
